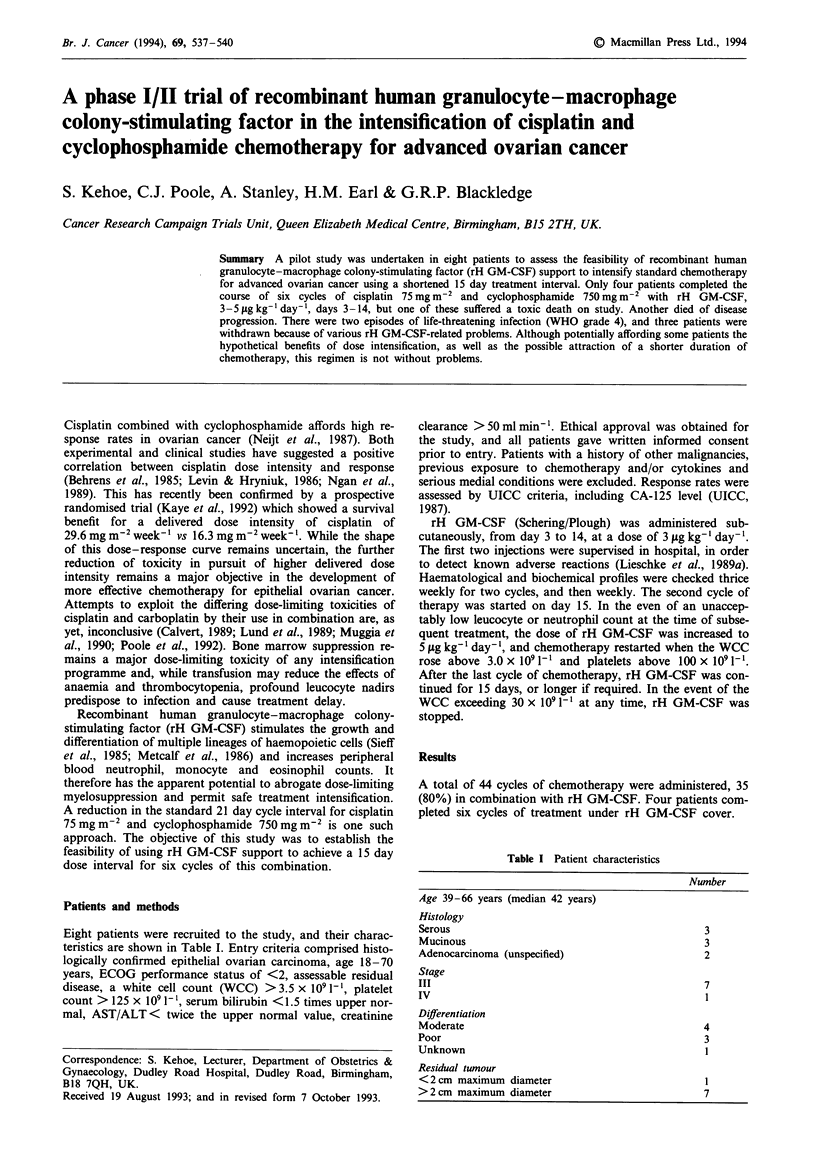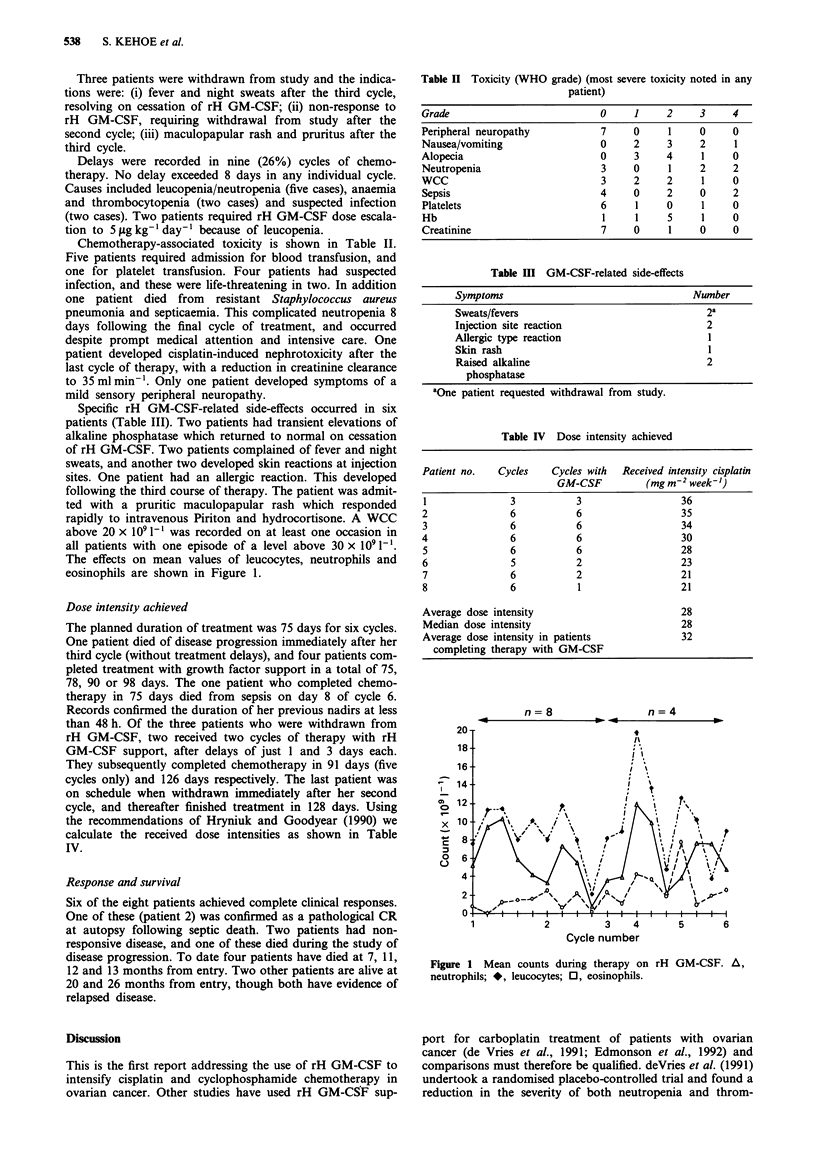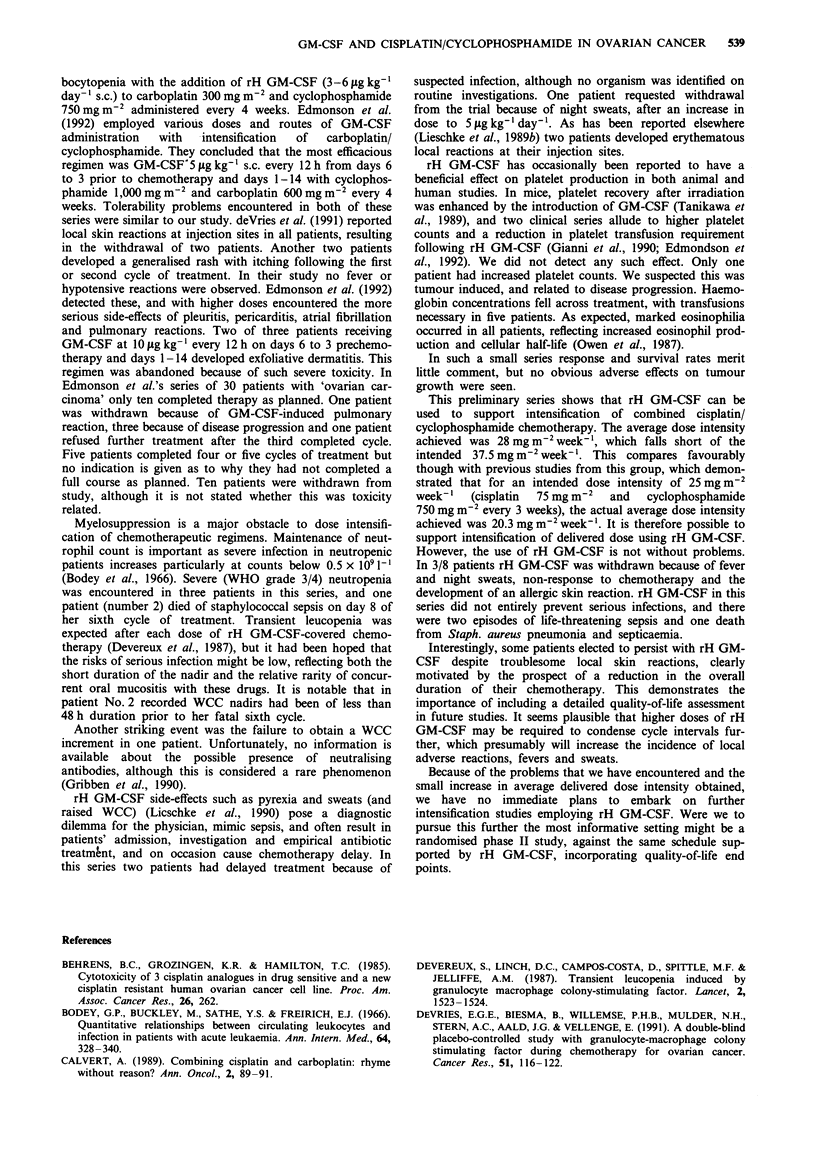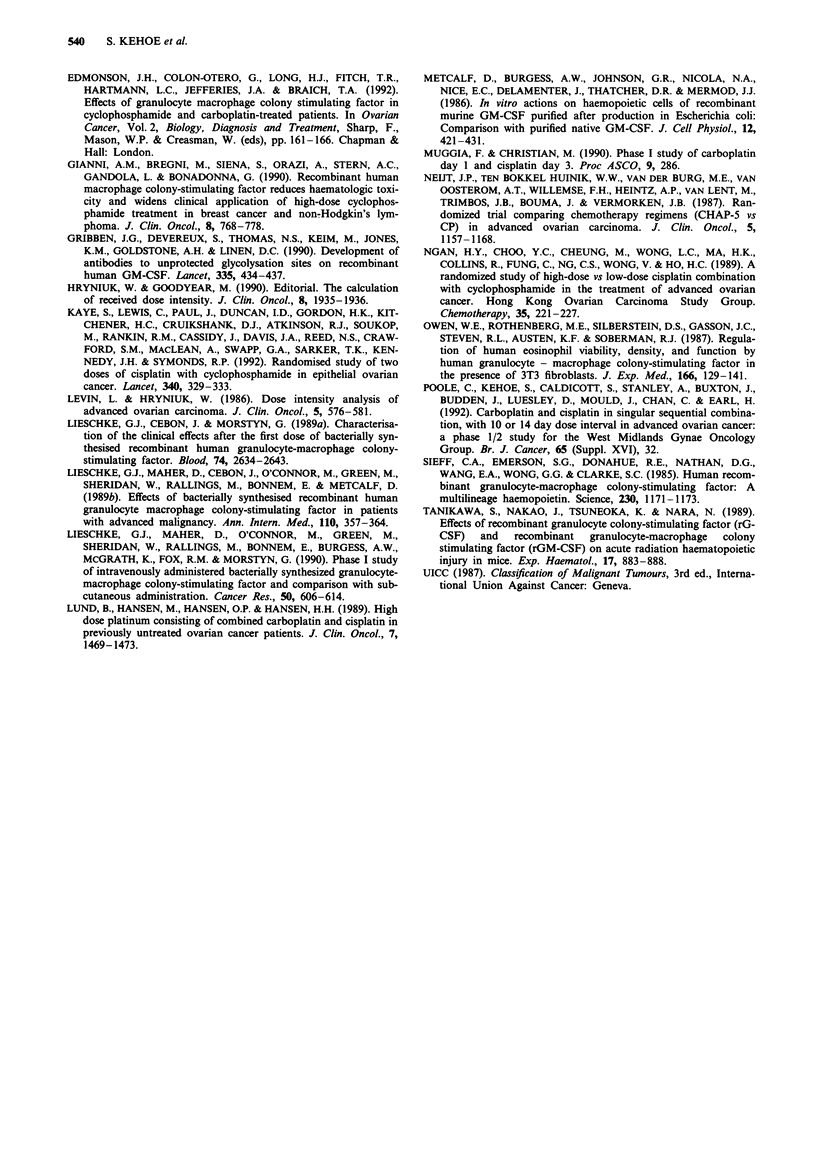# A phase I/II trial of recombinant human granulocyte-macrophage colony-stimulating factor in the intensification of cisplatin and cyclophosphamide chemotherapy for advanced ovarian cancer.

**DOI:** 10.1038/bjc.1994.97

**Published:** 1994-03

**Authors:** S. Kehoe, C. J. Poole, A. Stanley, H. M. Earl, G. R. Blackledge

**Affiliations:** Cancer Research Campaign Trials Unit, Queen Elizabeth Medical Centre, Birmingham, UK.

## Abstract

A pilot study was undertaken in eight patients to assess the feasibility of recombinant human granulocyte-macrophage colony-stimulating factor (rH GM-CSF) support to intensify standard chemotherapy for advanced ovarian cancer using a shortened 15 day treatment interval. Only four patients completed the course of six cycles of cisplatin 75 mg m-2 and cyclophosphamide 750 mg m-2 with rH GM-CSF, 3-5 micrograms kg-1 day-1, days 3-14, but one of these suffered a toxic death on study. Another died of disease progression. There were two episodes of life-threatening infection (WHO grade 4), and three patients were withdrawn because of various rH GM-CSF-related problems. Although potentially affording some patients the hypothetical benefits of dose intensification, as well as the possible attraction of a shorter duration of chemotherapy, this regimen is not without problems.


Br. J. Cancer (1994), 69, 537-540 © Macmillan Press Ltd., 1994

### A phase I/II trial of recombinant human granulocyte–macrophage colony-stimulating factor in the intensification of cisplatin and cyclophosphamide chemotherapy for advanced ovarian cancer

S. Kehoe, C.J. Poole, A. Stanley, H.M. Earl & G.R.P. Blackledge

*Cancer Research Campaign Trials Unit, Queen Elizabeth Medical Centre, Birmingham, B15 2TH, UK.*

**Summary** A pilot study was undertaken in eight patients to assess the feasibility of recombinant human granulocyte–macrophage colony-stimulating factor (rH GM-CSF) support to intensify standard chemotherapy for advanced ovarian cancer using a shortened 15 day treatment interval. Only four patients completed the course of six cycles of cisplatin 75 mg m$^{-2}$ and cyclophosphamide 750 mg m$^{-2}$ with rH GM-CSF, 3–5 μg kg$^{-1}$ day$^{-1}$, days 3–14, but one of these suffered a toxic death on study. Another died of disease progression. There were two episodes of life-threatening infection (WHO grade 4), and three patients were withdrawn because of various rH GM-CSF-related problems. Although potentially affording some patients the hypothetical benefits of dose intensification, as well as the possible attraction of a shorter duration of chemotherapy, this regimen is not without problems.

Cisplatin combined with cyclophosphamide affords high response rates in ovarian cancer (Neijt *et al.*, 1987). Both experimental and clinical studies have suggested a positive correlation between cisplatin dose intensity and response (Behrens *et al.*, 1985; Levin & Hryniuk, 1986; Ngan *et al.*, 1989). This has recently been confirmed by a prospective randomised trial (Kaye *et al.*, 1992) which showed a survival benefit for a delivered dose intensity of cisplatin of 29.6 mg m$^{-2}$ week$^{-1}$ *vs* 16.3 mg m$^{-2}$ week$^{-1}$. While the shape of this dose–response curve remains uncertain, the further reduction of toxicity in pursuit of higher delivered dose intensity remains a major objective in the development of more effective chemotherapy for epithelial ovarian cancer. Attempts to exploit the differing dose-limiting toxicities of cisplatin and carboplatin by their use in combination are, as yet, inconclusive (Calvert, 1989; Lund *et al.*, 1989; Muggia *et al.*, 1990; Poole *et al.*, 1992). Bone marrow suppression remains a major dose-limiting toxicity of any intensification programme and, while transfusion may reduce the effects of anaemia and thrombocytopenia, profound leucocyte nadirs predispose to infection and cause treatment delay.

Recombinant human granulocyte–macrophage colony-stimulating factor (rH GM-CSF) stimulates the growth and differentiation of multiple lineages of haemopoietic cells (Sieff *et al.*, 1985; Metcalf *et al.*, 1986) and increases peripheral blood neutrophil, monocyte and eosinophil counts. It therefore has the apparent potential to abrogate dose-limiting myelosuppression and permit safe treatment intensification. A reduction in the standard 21 day cycle interval for cisplatin 75 mg m$^{-2}$ and cyclophosphamide 750 mg m$^{-2}$ is one such approach. The objective of this study was to establish the feasibility of using rH GM-CSF support to achieve a 15 day dose interval for six cycles of this combination.

### Patients and methods

Eight patients were recruited to the study, and their characteristics are shown in Table I. Entry criteria comprised histologically confirmed epithelial ovarian carcinoma, age 18–70 years, ECOG performance status of <2, assessable residual disease, a white cell count (WCC) $>3.5\times10^9\,l^{-1}$, platelet count $>125\times10^9\,l^{-1}$, serum bilirubin <1.5 times upper normal, AST/ALT< twice the upper normal value, creatinine clearance >50 ml min$^{-1}$. Ethical approval was obtained for the study, and all patients gave written informed consent prior to entry. Patients with a history of other malignancies, previous exposure to chemotherapy and/or cytokines and serious medial conditions were excluded. Response rates were assessed by UICC criteria, including CA-125 level (UICC, 1987).

rH GM-CSF (Schering/Plough) was administered subcutaneously, from day 3 to 14, at a dose of 3 μg kg$^{-1}$ day$^{-1}$. The first two injections were supervised in hospital, in order to detect known adverse reactions (Lieschke *et al.*, 1989*a*). Haematological and biochemical profiles were checked thrice weekly for two cycles, and then weekly. The second cycle of therapy was started on day 15. In the even of an unacceptably low leucocyte or neutrophil count at the time of subsequent treatment, the dose of rH GM-CSF was increased to 5 μg kg$^{-1}$ day$^{-1}$, and chemotherapy restarted when the WCC rose above $3.0\times10^9\,l^{-1}$ and platelets above $100\times10^9\,l^{-1}$. After the last cycle of chemotherapy, rH GM-CSF was continued for 15 days, or longer if required. In the event of the WCC exceeding $30\times10^9\,l^{-1}$ at any time, rH GM-CSF was stopped.

### Results

A total of 44 cycles of chemotherapy were administered, 35 (80%) in combination with rH GM-CSF. Four patients completed six cycles of treatment under rH GM-CSF cover.

**Table I** Patient characteristics

| | *Number* |
|---|---|
| *Age* 39–66 years (median 42 years) | |
| *Histology* | |
| Serous | 3 |
| Mucinous | 3 |
| Adenocarcinoma (unspecified) | 2 |
| *Stage* | |
| III | 7 |
| IV | 1 |
| *Differentiation* | |
| Moderate | 4 |
| Poor | 3 |
| Unknown | 1 |
| *Residual tumour* | |
| <2 cm maximum diameter | 1 |
| >2 cm maximum diameter | 7 |

Correspondence: S. Kehoe, Lecturer, Department of Obstetrics & Gynaecology, Dudley Road Hospital, Dudley Road, Birmingham, B18 7QH, UK.
Received 19 August 1993; and in revised form 7 October 1993.

Three patients were withdrawn from study and the indications were: (i) fever and night sweats after the third cycle, resolving on cessation of rH GM-CSF; (ii) non-response to rH GM-CSF, requiring withdrawal from study after the second cycle; (iii) maculopapular rash and pruritus after the third cycle.

Delays were recorded in nine (26%) cycles of chemotherapy. No delay exceeded 8 days in any individual cycle. Causes included leucopenia/neutropenia (five cases), anaemia and thrombocytopenia (two cases) and suspected infection (two cases). Two patients required rH GM-CSF dose escalation to $5\,\mu g\,kg^{-1}\,day^{-1}$ because of leucopenia.

Chemotherapy-associated toxicity is shown in Table II. Five patients required admission for blood transfusion, and one for platelet transfusion. Four patients had suspected infection, and these were life-threatening in two. In addition one patient died from resistant *Staphylococcus aureus* pneumonia and septicaemia. This complicated neutropenia 8 days following the final cycle of treatment, and occurred despite prompt medical attention and intensive care. One patient developed cisplatin-induced nephrotoxicity after the last cycle of therapy, with a reduction in creatinine clearance to $35\,ml\,min^{-1}$. Only one patient developed symptoms of a mild sensory peripheral neuropathy.

Specific rH GM-CSF-related side-effects occurred in six patients (Table III). Two patients had transient elevations of alkaline phosphatase which returned to normal on cessation of rH GM-CSF. Two patients complained of fever and night sweats, and another two developed skin reactions at injection sites. One patient had an allergic reaction. This developed following the third course of therapy. The patient was admitted with a pruritic maculopapular rash which responded rapidly to intravenous Piriton and hydrocortisone. A WCC above $20 \times 10^9\,l^{-1}$ was recorded on at least one occasion in all patients with one episode of a level above $30 \times 10^9\,l^{-1}$. The effects on mean values of leucocytes, neutrophils and eosinophils are shown in Figure 1.

### *Dose intensity achieved*

The planned duration of treatment was 75 days for six cycles. One patient died of disease progression immediately after her third cycle (without treatment delays), and four patients completed treatment with growth factor support in a total of 75, 78, 90 or 98 days. The one patient who completed chemotherapy in 75 days died from sepsis on day 8 of cycle 6. Records confirmed the duration of her previous nadirs at less than 48 h. Of the three patients who were withdrawn from rH GM-CSF, two received two cycles of therapy with rH GM-CSF support, after delays of just 1 and 3 days each. They subsequently completed chemotherapy in 91 days (five cycles only) and 126 days respectively. The last patient was on schedule when withdrawn immediately after her second cycle, and thereafter finished treatment in 128 days. Using the recommendations of Hryniuk and Goodyear (1990) we calculate the received dose intensities as shown in Table IV.

### *Response and survival*

Six of the eight patients achieved complete clinical responses. One of these (patient 2) was confirmed as a pathological CR at autopsy following septic death. Two patients had non-responsive disease, and one of these died during the study of disease progression. To date four patients have died at 7, 11, 12 and 13 months from entry. Two other patients are alive at 20 and 26 months from entry, though both have evidence of relapsed disease.

**Table II** Toxicity (WHO grade) (most severe toxicity noted in any patient)

| *Grade* | *0* | *1* | *2* | *3* | *4* |
|---|---|---|---|---|---|
| Peripheral neuropathy | 7 | 0 | 1 | 0 | 0 |
| Nausea/vomiting | 0 | 2 | 3 | 2 | 1 |
| Alopecia | 0 | 3 | 4 | 1 | 0 |
| Neutropenia | 3 | 0 | 1 | 2 | 2 |
| WCC | 3 | 2 | 2 | 1 | 0 |
| Sepsis | 4 | 0 | 2 | 0 | 2 |
| Platelets | 6 | 1 | 0 | 1 | 0 |
| Hb | 1 | 1 | 5 | 1 | 0 |
| Creatinine | 7 | 0 | 1 | 0 | 0 |

**Table III** GM-CSF-related side-effects

| *Symptoms* | *Number* |
|---|---|
| Sweats/fevers | 2[a] |
| Injection site reaction | 2 |
| Allergic type reaction | 1 |
| Skin rash | 1 |
| Raised alkaline phosphatase | 2 |

[a]One patient requested withdrawal from study.

**Table IV** Dose intensity achieved

| *Patient no.* | *Cycles* | *Cycles with GM-CSF* | *Received intensity cisplatin ($mg\,m^{-2}\,week^{-1}$)* |
|---|---|---|---|
| 1 | 3 | 3 | 36 |
| 2 | 6 | 6 | 35 |
| 3 | 6 | 6 | 34 |
| 4 | 6 | 6 | 30 |
| 5 | 6 | 6 | 28 |
| 6 | 5 | 2 | 23 |
| 7 | 6 | 2 | 21 |
| 8 | 6 | 1 | 21 |
| Average dose intensity | | | 28 |
| Median dose intensity | | | 28 |
| Average dose intensity in patients completing therapy with GM-CSF | | | 32 |

**Figure 1** Mean counts during therapy on rH GM-CSF. Δ, neutrophils; ◆, leucocytes; □, eosinophils.

### Discussion

This is the first report addressing the use of rH GM-CSF to intensify cisplatin and cyclophosphamide chemotherapy in ovarian cancer. Other studies have used rH GM-CSF support for carboplatin treatment of patients with ovarian cancer (de Vries *et al.*, 1991; Edmonson *et al.*, 1992) and comparisons must therefore be qualified. deVries *et al.* (1991) undertook a randomised placebo-controlled trial and found a reduction in the severity of both neutropenia and thrombocytopenia with the addition of rH GM-CSF (3–6 $\mu g\,kg^{-1}\,day^{-1}$ s.c.) to carboplatin 300 $mg\,m^{-2}$ and cyclophosphamide 750 $mg\,m^{-2}$ administered every 4 weeks. Edmonson *et al.* (1992) employed various doses and routes of GM-CSF administration with intensification of carboplatin/ cyclophosphamide. They concluded that the most efficacious regimen was GM-CSF 5 $\mu g\,kg^{-1}$ s.c. every 12 h from days 6 to 3 prior to chemotherapy and days 1–14 with cyclophosphamide 1,000 $mg\,m^{-2}$ and carboplatin 600 $mg\,m^{-2}$ every 4 weeks. Tolerability problems encountered in both of these series were similar to our study. deVries *et al.* (1991) reported local skin reactions at injection sites in all patients, resulting in the withdrawal of two patients. Another two patients developed a generalised rash with itching following the first or second cycle of treatment. In their study no fever or hypotensive reactions were observed. Edmonson *et al.* (1992) detected these, and with higher doses encountered the more serious side-effects of pleuritis, pericarditis, atrial fibrillation and pulmonary reactions. Two of three patients receiving GM-CSF at 10 $\mu g\,kg^{-1}$ every 12 h on days 6 to 3 prechemotherapy and days 1–14 developed exfoliative dermatitis. This regimen was abandoned because of such severe toxicity. In Edmonson *et al.*'s series of 30 patients with 'ovarian carcinoma' only ten completed therapy as planned. One patient was withdrawn because of GM-CSF-induced pulmonary reaction, three because of disease progression and one patient refused further treatment after the third completed cycle. Five patients completed four or five cycles of treatment but no indication is given as to why they had not completed a full course as planned. Ten patients were withdrawn from study, although it is not stated whether this was toxicity related.

Myelosuppression is a major obstacle to dose intensification of chemotherapeutic regimens. Maintenance of neutrophil count is important as severe infection in neutropenic patients increases particularly at counts below $0.5 \times 10^9\,l^{-1}$ (Bodey *et al.*, 1966). Severe (WHO grade 3/4) neutropenia was encountered in three patients in this series, and one patient (number 2) died of staphylococcal sepsis on day 8 of her sixth cycle of treatment. Transient leucopenia was expected after each dose of rH GM-CSF-covered chemotherapy (Devereux *et al.*, 1987), but it had been hoped that the risks of serious infection might be low, reflecting both the short duration of the nadir and the relative rarity of concurrent oral mucositis with these drugs. It is notable that in patient No. 2 recorded WCC nadirs had been of less than 48 h duration prior to her fatal sixth cycle.

Another striking event was the failure to obtain a WCC increment in one patient. Unfortunately, no information is available about the possible presence of neutralising antibodies, although this is considered a rare phenomenon (Gribben *et al.*, 1990).

rH GM-CSF side-effects such as pyrexia and sweats (and raised WCC) (Licschke *et al.*, 1990) pose a diagnostic dilemma for the physician, mimic sepsis, and often result in patients' admission, investigation and empirical antibiotic treatment, and on occasion cause chemotherapy delay. In this series two patients had delayed treatment because of suspected infection, although no organism was identified on routine investigations. One patient requested withdrawal from the trial because of night sweats, after an increase in dose to 5 $\mu g\,kg^{-1}\,day^{-1}$. As has been reported elsewhere (Lieschke *et al.*, 1989*b*) two patients developed erythematous local reactions at their injection sites.

rH GM-CSF has occasionally been reported to have a beneficial effect on platelet production in both animal and human studies. In mice, platelet recovery after irradiation was enhanced by the introduction of GM-CSF (Tanikawa *et al.*, 1989), and two clinical series allude to higher platelet counts and a reduction in platelet transfusion requirement following rH GM-CSF (Gianni *et al.*, 1990; Edmondson *et al.*, 1992). We did not detect any such effect. Only one patient had increased platelet counts. We suspected this was tumour induced, and related to disease progression. Haemoglobin concentrations fell across treatment, with transfusions necessary in five patients. As expected, marked eosinophilia occurred in all patients, reflecting increased eosinophil production and cellular half-life (Owen *et al.*, 1987).

In such a small series response and survival rates merit little comment, but no obvious adverse effects on tumour growth were seen.

This preliminary series shows that rH GM-CSF can be used to support intensification of combined cisplatin/ cyclophosphamide chemotherapy. The average dose intensity achieved was 28 $mg\,m^{-2}\,week^{-1}$, which falls short of the intended 37.5 $mg\,m^{-2}\,week^{-1}$. This compares favourably though with previous studies from this group, which demonstrated that for an intended dose intensity of 25 $mg\,m^{-2}\,week^{-1}$ (cisplatin 75 $mg\,m^{-2}$ and cyclophosphamide 750 $mg\,m^{-2}$ every 3 weeks), the actual average dose intensity achieved was 20.3 $mg\,m^{-2}\,week^{-1}$. It is therefore possible to support intensification of delivered dose using rH GM-CSF. However, the use of rH GM-CSF is not without problems. In 3/8 patients rH GM-CSF was withdrawn because of fever and night sweats, non-response to chemotherapy and the development of an allergic skin reaction. rH GM-CSF in this series did not entirely prevent serious infections, and there were two episodes of life-threatening sepsis and one death from *Staph. aureus* pneumonia and septicaemia.

Interestingly, some patients elected to persist with rH GM-CSF despite troublesome local skin reactions, clearly motivated by the prospect of a reduction in the overall duration of their chemotherapy. This demonstrates the importance of including a detailed quality-of-life assessment in future studies. It seems plausible that higher doses of rH GM-CSF may be required to condense cycle intervals further, which presumably will increase the incidence of local adverse reactions, fevers and sweats.

Because of the problems that we have encountered and the small increase in average delivered dose intensity obtained, we have no immediate plans to embark on further intensification studies employing rH GM-CSF. Were we to pursue this further the most informative setting might be a randomised phase II study, against the same schedule supported by rH GM-CSF, incorporating quality-of-life end points.

### References

BEHRENS, B.C., GROZINGEN, K.R. & HAMILTON, T.C. (1985). Cytotoxicity of 3 cisplatin analogues in drug sensitive and a new cisplatin resistant human ovarian cancer cell line. *Proc. Am. Assoc. Cancer Res.*, **26**, 262.

BODEY, G.P., BUCKLEY, M., SATHE, Y.S. & FREIRICH, E.J. (1966). Quantitative relationships between circulating leukocytes and infection in patients with acute leukaemia. *Ann. Intern. Med.*, **64**, 328–340.

CALVERT, A. (1989). Combining cisplatin and carboplatin: rhyme without reason? *Ann. Oncol.*, **2**, 89–91.

DEVEREUX, S., LINCH, D.C., CAMPOS-COSTA, D., SPITTLE, M.F. & JELLIFFE, A.M. (1987). Transient leucopenia induced by granulocyte macrophage colony-stimulating factor. *Lancet*, **2**, 1523–1524.

DEVRIES, E.G.E., BIESMA, B., WILLEMSE, P.H.B., MULDER, N.H., STERN, A.C., AALD, J.G. & VELLENGE, E. (1991). A double-blind placebo-controlled study with granulocyte-macrophage colony stimulating factor during chemotherapy for ovarian cancer. *Cancer Res.*, **51**, 116–122.

EDMONSON, J.H., COLON-OTERO, G., LONG, H.J., FITCH, T.R., HARTMANN, L.C., JEFFERIES, J.A. & BRAICH, T.A. (1992). Effects of granulocyte macrophage colony stimulating factor in cyclophosphamide and carboplatin-treated patients. In *Ovarian Cancer*, Vol. 2, *Biology, Diagnosis and Treatment*, Sharp, F., Mason, W.P. & Creasman, W. (eds), pp. 161-166. Chapman & Hall: London.

GIANNI, A.M., BREGNI, M., SIENA, S., ORAZI, A., STERN, A.C., GANDOLA, L. & BONADONNA, G. (1990). Recombinant human macrophage colony-stimulating factor reduces haematologic toxicity and widens clinical application of high-dose cyclophosphamide treatment in breast cancer and non-Hodgkin's lymphoma. *J. Clin. Oncol.*, **8**, 768-778.

GRIBBEN, J.G., DEVEREUX, S., THOMAS, N.S., KEIM, M., JONES, K.M., GOLDSTONE, A.H. & LINEN, D.C. (1990). Development of antibodies to unprotected glycolysation sites on recombinant human GM-CSF. *Lancet*, **335**, 434-437.

HRYNIUK, W. & GOODYEAR, M. (1990). Editorial. The calculation of received dose intensity. *J. Clin. Oncol.*, **8**, 1935-1936.

KAYE, S., LEWIS, C., PAUL, J., DUNCAN, I.D., GORDON, H.K., KITCHENER, H.C., CRUIKSHANK, D.J., ATKINSON, R.J., SOUKOP, M., RANKIN, R.M., CASSIDY, J., DAVIS, J.A., REED, N.S., CRAWFORD, S.M., MACLEAN, A., SWAPP, G.A., SARKER, T.K., KENNEDY, J.H. & SYMONDS, R.P. (1992). Randomised study of two doses of cisplatin with cyclophosphamide in epithelial ovarian cancer. *Lancet*, **340**, 329-333.

LEVIN, L. & HRYNIUK, W. (1986). Dose intensity analysis of advanced ovarian carcinoma. *J. Clin. Oncol.*, **5**, 576-581.

LIESCHKE, G.J., CEBON, J. & MORSTYN, G. (1989*a*). Characterisation of the clinical effects after the first dose of bacterially synthesised recombinant human granulocyte-macrophage colony-stimulating factor. *Blood*, **74**, 2634-2643.

LIESCHKE, G.J., MAHER, D., CEBON, J., O'CONNOR, M., GREEN, M., SHERIDAN, W., RALLINGS, M., BONNEM, E. & METCALF, D. (1989*b*). Effects of bacterially synthesised recombinant human granulocyte macrophage colony-stimulating factor in patients with advanced malignancy. *Ann. Intern. Med.*, **110**, 357-364.

LIESCHKE, G.J., MAHER, D., O'CONNOR, M., GREEN, M., SHERIDAN, W., RALLINGS, M., BONNEM, E., BURGESS, A.W., MCGRATH, K., FOX, R.M. & MORSTYN, G. (1990). Phase I study of intravenously administered bacterially synthesized granulocyte-macrophage colony-stimulating factor and comparison with subcutaneous administration. *Cancer Res.*, **50**, 606-614.

LUND, B., HANSEN, M., HANSEN, O.P. & HANSEN, H.H. (1989). High dose platinum consisting of combined carboplatin and cisplatin in previously untreated ovarian cancer patients. *J. Clin. Oncol.*, **7**, 1469-1473.

METCALF, D., BURGESS, A.W., JOHNSON, G.R., NICOLA, N.A., NICE, E.C., DELAMENTER, J., THATCHER, D.R. & MERMOD, J.J. (1986). *In vitro* actions on haemopoietic cells of recombinant murine GM-CSF purified after production in Escherichia coli: Comparison with purified native GM-CSF. *J. Cell Physiol.*, **12**, 421-431.

MUGGIA, F. & CHRISTIAN, M. (1990). Phase I study of carboplatin day 1 and cisplatin day 3. *Proc ASCO*, **9**, 286.

NEIJT, J.P., TEN BOKKEL HUINIK, W.W., VAN DER BURG, M.E., VAN OOSTEROM, A.T., WILLEMSE, F.H., HEINTZ, A.P., VAN LENT, M., TRIMBOS, J.B., BOUMA, J. & VERMORKEN, J.B. (1987). Randomized trial comparing chemotherapy regimens (CHAP-5 *vs* CP) in advanced ovarian carcinoma. *J. Clin. Oncol.*, **5**, 1157-1168.

NGAN, H.Y., CHOO, Y.C., CHEUNG, M., WONG, L.C., MA, H.K., COLLINS, R., FUNG, C., NG, C.S., WONG, V. & HO, H.C. (1989). A randomized study of high-dose *vs* low-dose cisplatin combination with cyclophosphamide in the treatment of advanced ovarian cancer. Hong Kong Ovarian Carcinoma Study Group. *Chemotherapy*, **35**, 221-227.

OWEN, W.E., ROTHENBERG, M.E., SILBERSTEIN, D.S., GASSON, J.C., STEVEN, R.L., AUSTEN, K.F. & SOBERMAN, R.J. (1987). Regulation of human eosinophil viability, density, and function by human granulocyte - macrophage colony-stimulating factor in the presence of 3T3 fibroblasts. *J. Exp. Med.*, **166**, 129-141.

POOLE, C., KEHOE, S., CALDICOTT, S., STANLEY, A., BUXTON, J., BUDDEN, J., LUESLEY, D., MOULD, J., CHAN, C. & EARL, H. (1992). Carboplatin and cisplatin in singular sequential combination, with 10 or 14 day dose interval in advanced ovarian cancer: a phase 1/2 study for the West Midlands Gynae Oncology Group. *Br. J. Cancer*, **65** (Suppl. XVI), 32.

SIEFF, C.A., EMERSON, S.G., DONAHUE, R.E., NATHAN, D.G., WANG, E.A., WONG, G.G. & CLARKE, S.C. (1985). Human recombinant granulocyte-macrophage colony-stimulating factor: A multilineage haemopoietin. Science, **230**, 1171-1173.

TANIKAWA, S., NAKAO, J., TSUNEOKA, K. & NARA, N. (1989). Effects of recombinant granulocyte colony-stimulating factor (rG-CSF) and recombinant granulocyte-macrophage colony stimulating factor (rGM-CSF) on acute radiation haematopoietic injury in mice. *Exp. Haematol.*, **17**, 883-888.

UICC (1987). *Classification of Malignant Tumours*, 3rd ed., International Union Against Cancer: Geneva.